# Disrupting resilient criminal networks through data analysis: The case of Sicilian Mafia

**DOI:** 10.1371/journal.pone.0236476

**Published:** 2020-08-05

**Authors:** Lucia Cavallaro, Annamaria Ficara, Pasquale De Meo, Giacomo Fiumara, Salvatore Catanese, Ovidiu Bagdasar, Wei Song, Antonio Liotta

**Affiliations:** 1 University of Derby, Derby, United Kingdom; 2 University of Palermo, Palermo, Italy; 3 University of Messina, Polo Universitario Annunziata, Messina, Italy; 4 MIFT Department, University of Messina, Messina, Italy; 5 Shanghai Ocean University, Shanghai, China; 6 Edinburgh Napier University, Edinburgh, United Kingdom; Universite Catholique de Louvain, BELGIUM

## Abstract

Compared to other types of social networks, criminal networks present particularly hard challenges, due to their strong resilience to disruption, which poses severe hurdles to Law-Enforcement Agencies (LEAs). Herein, we borrow methods and tools from Social Network Analysis (SNA) to (i) unveil the structure and organization of Sicilian Mafia gangs, based on two real-world datasets, and (ii) gain insights as to how to efficiently reduce the Largest Connected Component (LCC) of two networks derived from them. Mafia networks have peculiar features in terms of the links distribution and strength, which makes them very different from other social networks, and extremely robust to exogenous perturbations. Analysts also face difficulties in collecting reliable datasets that accurately describe the gangs’ internal structure and their relationships with the external world, which is why earlier studies are largely qualitative, elusive and incomplete. An added value of our work is the generation of two real-world datasets, based on raw data extracted from juridical acts, relating to a Mafia organization that operated in Sicily during the first decade of 2000s. We created two different networks, capturing phone calls and physical meetings, respectively. Our analysis simulated different intervention procedures: (i) arresting one criminal at a time (sequential node removal); and (ii) police raids (node block removal). In both the sequential, and the node block removal intervention procedures, the Betweenness centrality was the most effective strategy in prioritizing the nodes to be removed. For instance, when targeting the top 5% nodes with the largest Betweenness centrality, our simulations suggest a reduction of up to 70% in the size of the LCC. We also identified that, due the peculiar type of interactions in criminal networks (namely, the distribution of the interactions’ frequency), no significant differences exist between weighted and unweighted network analysis. Our work has significant practical applications for perturbing the operations of criminal and terrorist networks.

## Introduction

The Sicilian Mafia, known as *Cosa Nostra*, is a specific type of criminal organization. It began in Sicily and then grew into a major international criminal organization [1–3], taking control and influencing economic, social and political sectors of entire countries. Compared to other criminal organizations, Mafia has a unique structure: it appears as a collection of loosely coupled groups, which last for several generations [3, 4]. Each of these groups can be referred to as *cosca* (i.e., a Sicilian word which refers to any plant whose spiny closely folded leaves symbolize the tightness of relationships between members of the Mafia), *gang*, *clan* or *family*. Members of a Mafia gang are tied by strong links, thus making the organization efficient and capable of swiftly re-organizing itself to pursue the most profitable activities and adjust to law enforcement operations. In fact, Mafia tends to create deep roots into the very fabric of society, to the point that it becomes “impossible to destroy without a radical change in social institutions” (in the words of Italian politician Leopoldo Franchetti, 1876 [1]).

An impressive scientific interest for the study of social structure of Sicilian Mafia syndicates has been generated because of the social embeddedness of this type of criminal organization [5, 6]. Herein, we borrow methods and tools from Social Network Analysis (SNA) to (i) unveil the structure and organization of Sicilian Mafia gangs, based on two real-world datasets, and (ii) gain insights as to how to reduce the size of the Largest Connected Component (LCC) of two real Sicilian Mafia networks.

In graph theory, the LCC is a way to measure the network’s connectivity. Its size defines how many people a single individual (i.e., a node) is able to reach through its relationship bonds (i.e., direct links/edges or paths). Indeed, if the LCC size has the same order of magnitude of the network size, then the connectivity is at its maximum. On the other hand, nodes removal (jointly with their links) may provoke the emerging of smaller clusters (or even isolated nodes). As a consequence, a LCC size drop implies that the network becomes less and less connected. Thus, the LCC size allows to quantify the effectiveness of the nodes removal strategies.

SNA is typically employed by Law Enforcement Agencies (LEAs) to analyze criminal networks, investigate the relations among criminals, and evaluate the effectiveness of law enforcement interventions aimed at disrupting criminal networks [7]. Morselli [8] studied the connections within the Gambino, New York based family, and he focused on the career of Saul Gravano, a member of the Gambino family. McGloin [2] made an analysis on the structure of a network based on the street gangs in Newark, New Jersey. Calderoni [9] explained how illicit drug traffics were indirectly handled by high-status Mafia members, whereas the most central and visible positions were held by middle-level criminals.

On the other hand, when seeking optimal disruptive strategies for criminal networks, two main approaches can be considered [10]: the *human capital* and the *social capital*. The former originates from economics, and refers to the personal attributes and/or resources possessed by actors within a social network. Sparrow [11] suggested that identifying the individuals who possess many resources and skills offers a great opportunity to damage the criminal network. Cornish [12] introduced the notion of *script*, which is borrowed from cognitive science. A script approach is a way to better understand how crimes are committed and how to prevent them. The central element of this approach, the crime script, is a step-by-step account of the actions and decisions involved in a crime. If the script is correctly identified, it can be used to prevent or disrupt crime commission. Later on, Bruinsma and Bernasco [13] combined this script concept with SNA, to identify the role of human capital within criminal networks.

By contrast, the social capital network-disruption strategy [14, 15] refers to the connections or ties between the actors in a network. It is through these connections that actors can have strategic positions, exchanging and sharing resources with other actors in the network [10, 11, 16–18]. Research in this field is often based on SNA to find the most influential or powerful individuals of social capital, who correspond to the most central nodes in a network [19]. There is empirical evidence that *brokers* (i.e., the individuals acting as bridges between disconnected subgroups) have a key role in the connectivity of criminal networks, often relating separate criminal collectives within illegal markets [20, 17, 21–26]. For instance, the impact of brokers on the crime commission processes has been investigated by Morselli and Roy [20].

Through SNA, a number of interesting findings have been made over the years. Agreste *et al*. [26] devised an efficient approch for dismantling mafia syndicates, based on the application of percolation theory. Peterson [27] argued that the most central actors in covert networks might also be the most visible, and for this reason the most likely to be detected. Spapens [28] identified a brokerage role within Dutch ecstasy production, observing that brokers not only increase “social capital” within these criminal collectives, but also add “human capital”. Bright *et al*. [29] investigated the effectiveness of five law enforcement interventions in disrupting and dismantling criminal networks, using both the social, and human capital approaches. Moreover, they showed how the removal of actors based on the Betweenness centrality metric was the most efficient strategy.

In this paper we focus particularly on one aspect of *network resilience*, that is the ability of criminal networks to survive the actions of the LEAs. However, network resilience also relates to the capacity of these networks to reorganize after perturbations (e.g., police raids), and to reestablish connectivity. This latter aspect has not been investigated herein because our networks are currently treated as a static dataset. Nonetheless, this work is a significantly extended version of the early proof-of-concept results published in our recent conference paper [30].

Several authors [31, 32] have described the concept of network resilience considering two main aspects: (i) the capacity to absorb and thus resist disruption, (ii) the capacity to modify the network’s internal structure and strategies, in order to adapt to external pressures. Resilience depends on the level of *redundancy* present in the criminal network. Redundancy is reflected in the number of relationships among the network actors, and is associated with strong connections between these actors [33]. A high level of redundancy and consequently of diversity of relationships in the network allows it to function even if some tie is broken and to find more options to substitute the network actors who have been arrested, incarcerated, or killed by LEAs. Replacements are often found within short social distances because criminal connections often start from already established social networks of kinship, friendship, or emotional ties [5, 34].

We have mentioned that the Sicilian Mafia differs from other criminal organizations. Mafia networks have peculiar features, due to the links distribution and strength, which makes them extremely robust to exogenous perturbations. Analysts are also faced with the difficulty in collecting reliable datasets that accurately describe the gangs’ internal structure and their relationships with the external world, which is why earlier studies are largely qualitative, elusive and incomplete. A key feature of our work is the generation of two real-world datasets, which we have anonymized (to eliminate sensitive data) and made publicly available online on GitHub repository (https://github.com/lcucav/criminal-nets/tree/master/disruption) and on Zenodo (https://doi.org/10.5281/zenodo.3938818). These are based on raw data derived from juridical acts, relating to a Mafia gang that operated in Sicily (Italy) during the first decade of 2000s. We have chosen the court order associated with the “Montagna” operation because it contained the largest number of wiretaps and stakeout among all court orders we could manage. Consequently, we were able to set up a much larger dataset than what we could have obtained by focusing on other court orders. We created two very different networks, capturing phone calls and physical meetings, respectively, which we have characterized in our recent conference paper [35].

Our datasets relate to a Mafia syndicate acting as a link between prominent criminal families, operating in the two biggest cities (Palermo and Catania) of Southern Italy. The Phone Calls dataset has been derived from eavesdropping, while the Meetings dataset has been derived from police surveillance data. Both datasets are represented by undirected networks. For each of them we have created a weighted graph version (considering the frequency of interactions between individuals, or nodes), as well as an unweighted version (accounting only for connections).

The present study goes well beyond the initial characterization of these datasets [35], investigating network robustness across different scenarios, pinpointing the most effective metric, and demonstrating an effective strategy to obtain a faster LCC size drop. We simulate two types of police operations: (i) arresting one criminal at a time (sequential node removal), and (ii) police raids (node block removal). We evaluate how the different types of networks are impacted by these two types of perturbations, in terms of LCC size drop.

Generally, the resilience of a network is the result of several factors deriving from the network structure, such as the position of the nodes (i.e., the social capital), and individual technical abilities (i.e., the human capital). The former refers to the most connected actors (i.e., key players) of a criminal network and/or the nodes connecting the several groups and subgroups inside a network (i.e., bridges). The latter refers to the precious knowledge, skills and technical abilities of a criminal network node (e.g., pharmacological and chemical knowledge are required in drug synthesis processes). Villani *et al*. [36] tried to check whether, and to which extent, strategies based on both human and social capital could reduce (or neutralize) resistance and adaptation abilities of criminal organizations. Thus, we base our attack strategy on the social capital approach. We employ SNA methods to identify the actors having a high level of social capital. These are typically the most influential individuals, with a *central* role in the criminal network. To this end, we put to test four different centrality metrics, namely: (i) *Degree centrality*, (ii) *Betweenness centrality*, (iii) *Katz centrality*, and (iv) *Collective Influence*. It is worth recalling from SNA that the Degree centrality helps identifying network hubs (i.e., the focal points). However, contrary to other types of networks, criminal networks communications are not necessarily mediated via hubs, which are more visible and, thus, more vulnerable [21, 27]. On the other hand, by weighing the communication paths (rather than nodes in isolation), Betweeness centrality pinpoints those nodes that play an important role in multiple communication paths. We have therefore hypothesized that Betweeness centrality could help removing the individuals that are crucial in maintaining the information network. In turn, removing those individuals would increase the LCC size drop inside the networks, which is our aim.

For the sake of completeness, we also considered two more prominent centrality metrics. Katz centrality computes the relative influence of a node, measuring the number of node’s immediate neighbours (first degree nodes) and also all the other nodes in the network that connect to the node itself through these immediate neighbours. Finally, Collective Influence establishes the centrality of a node in a criminal network taking into account the degree of the node’s neighbours at a given distance *l* from it.

Our analysis identifies Betweenness centrality as the most effective metric, showing how, by neutralizing only 5% of the affiliates, the LCC size dropped by 70%. We also identified that, due the peculiar type of interactions in criminal networks (namely, the distribution of the interactions frequency) no significant differences exist between weighted and unweighted network analysis. Our work has significant practical applications for tackling criminal and terrorist networks.

## Materials and methods

### Background

In this paper we consider two weighted and undirected criminal networks or graphs. For convenience, we first summarise some key concepts of network theory [37] and formalize the problem at hand.

An *unweighted graph G* = (*V*, *E*) consists of a finite set *V* of *n* vertices (also called nodes or actors), and a set *E* ⊆ *V* × *V* of edges (also called links or ties).

A *weighted graph* is defined as a triplet *G* = (*V*, *E*, *W*), consisting of a finite set of vertices *V*, a set of edges *E* ⊆ *V* × *V* between nodes, and a set of positive weights *W*: *E* → *R*_++_ defined on each edge.

When all the edges are bidirectional, the graph is called *undirected*.

A *path* from node *u* to *v* is a sequence of nodes starting with *u* and ending with *v*, such that between any consecutive nodes there is a link. The *path cost* is the sum of the weights of all links along the path. A *shortest path* from *u* to *v* is a path along which the path cost is minimal (there may exist multiple shortest paths). In weighted graphs, the path cost equals is sum of the weights along the path. For unweighted graphs, a shortest path is that (or those) consisting of the fewest number of links.

A *connected component* [38] of an undirected graph is a subgraph in which any two vertices are connected to each other by paths, and which is connected to no additional vertices outside of this component (i.e., the connected components represent a partition of the whole graph, sometimes called *supergraph*). The LCC is the biggest one among all the connected components in a graph.

The *adjacency matrix* of a graph *G* defined over the set of vertices *V* = {1, …, *n*}, is an *n* × *n* square matrix denoted by *A* = (*a*_*ij*_), 1 ≤ *i*, *j* ≤ *n*, where *a*_*ij*_ = 1 if there exists an edge joining vertices *i* and *j*, and *a*_*ij*_ = 0 otherwise. Moreover, if the element (*i*, *j*) of the matrix *A*^*k*^ (*k* ≥ 2) is one, then the nodes *i* and *j* are connected through some walk of length exactly *k*. The weighted networks can be represented by giving the elements of the adjacency matrix values equal to the weights of the corresponding connections.

*Centrality* is a key concept in network analysis, and refers to the importance of a node in a network. There are multiple measures for network centrality in use within SNA, but the two most common centrality measures relating to strategic positions are Degree centrality and Betweenness centrality [11, 16].

*Degree centrality* [39] is a measure which evaluates the local importance of a node within the graph; given a node *i*, the Degree centrality *C*_*D*_(*i*) of *i* is defined by:

$$\begin{array}{c} C_{D}\left(i\right)=\sum_{j\in V,j\neq i}a_{ij}, \end{array}$$ (1)

where *A* = (*a*_*ij*_) is the adjacency matrix of the graph.

The *node degree* represents the number of edges adjacent to the node, while the *weighted node degree* is the sum of the edge weights, for edges incident to that node. This measure has been formalized as follows [40]:

$$\begin{array}{c} C_{D}^{W}\left(i\right)=\sum_{j\in V,(i,j)\in E}w_{ij}, \end{array}$$ (2)

where *w* is the weighted adjacency matrix, in which *w*_*ij*_ is greater than 0 if the node *i* is connected to node *j*, and the value represents the weight of the edge.

Some nodes may play a particularly important role in propagating information because they act as bridges between separate regions of a graph and so they have the potential to slow down (or magnify) the information flow from one region to another. Such nodes are said to have a high value of *Betweenness centrality* [41]. Specifically, the (shortest-path) betweenness *C*_*B*_(*i*) of a node *i* is defined as follows:

$$\begin{array}{c} C_{B}\left(i\right)=\sum_{s,t\in V,s\neq t}\frac{\sigma (s,t|i)}{\sigma (s,t)}, \end{array}$$ (3)

where *σ*(*s*, *t*) is the number of shortest paths between an arbitrary pair of nodes *s* and *t*, while *σ*(*s*, *t* ∣ *i*) denotes those shortest paths passing through the node *i*.

Betweenness centrality is a measure based on shortest paths for both unweighted and weighted networks. For our algorithm, we use Breadth-First Search (BFS) for unweighted and Dijkstra’s algorithm for weighted graphs.

*Katz centrality* [42] is another centrality measure, which defines the centrality for a node based on the centrality of its neighbours. For a node *i* this is defined as:

$$\begin{array}{c} C_{K}\left(i\right)=\alpha \sum_{j\in V}a_{ij}C_{K}\left(j\right)+\beta , \end{array}$$ (4)

where *α* and *β* are positive constants, *A* = (*a*_*ij*_) is the adjacency matrix of the graph, whose eigenvalues are denoted by λ_*i*_, *i* = 1, …, *n*. The parameter *β* controls the initial centrality, while the parameter *α* satisfies the inequality:

$$\begin{array}{c} \alpha <\frac{1}{\text{max}\{\lambda_{i}:\;1\leq i\leq n\}}. \end{array}$$

The Katz centrality for weighted networks can be computed in a similar way, but in this case we have to use the weighted adjacency matrix instead.

Another useful network metric is the *Collective Influence* (CI) [43], which computes the centrality (or influence) of a node *i* of a network according to the formula:

$$\begin{array}{c} {\text{CI}}_{\ell }\left(i\right)=\left(k_{i}-1\right)\sum_{j\in \delta B(i,\ell )}\left(k_{j}-1\right), \end{array}$$ (5)

where *k*_*i*_ is the degree of node *i*, *B*(*i*, *ℓ*) is the ball of radius *ℓ* centered on node *i*, and *δB*(*i*, *ℓ*) is the frontier of the ball, that is, the set of nodes at distance *ℓ* from *i* (the distance between two nodes is defined as the number of edges of the shortest path connecting them). To compute *CI*_*#x2113;*_(*i*), we first find the nodes on the frontier *δB*(*i*, *ℓ*). To compute the CI in a weighted network, we have to substitute the degree *k* of a node by his weighted degree given by formula (2).

*Clustering Coefficient* (CC) is a measure of the degree to which nodes in a graph tend to cluster together. For unweighted graphs, the clustering of a node *i*, denoted by *CC*(*i*), is the fraction of possible triangles through that node that exist, given by

$$\begin{array}{c} \text{CC}\left(i\right)=\frac{2T(i)}{k_{i}\left(k_{i}-1\right)}, \end{array}$$ (6)

where *T*(*i*) is the number of triangles through node *i* and *k*_*i*_ is the degree of *i*. For weighted graphs, the clustering is defined as the geometric average of the subgraph edge weights [44], obtained from the formula

$$\begin{array}{c} \text{CC}\left(i\right)=\frac{1}{k_{i}\left(k_{i}-1\right)}\sum_{i,j\in V}{\left({\hat{w}}_{ij}{\hat{w}}_{iw}{\hat{w}}_{jw}\right)}^{1/3}, \end{array}$$ (7)

where ${\hat{w}}_{ij}$ are the edge weights normalized by the maximum weight in the network ${\hat{w}}_{ij}=w_{ij}/\text{max}\left(w\right)$.

The *Average Path Length* (APL) [45] is a useful metric for assessing the connectivity of a graph. It is the average number of steps along the shortest paths for all possible pairs of network nodes. The average path length is defined as follows:

$$\begin{array}{c} \text{APL}=\sum_{i,j\in V}\frac{d(i,j)}{n(n-1)} \end{array}$$ (8)

where *V* = {1, …, *n*} is the set of nodes in the graph *G*, *d*(*i*, *j*) is the length of the shortest path from node *i* to node *j*, and *n* is the number of nodes in *G*.

### Dataset collection

In this section we describe how the datasets have been collected. Our datasets were derived from the pre-trial detention order, issued by the Court of Messina’s preliminary investigation judge on March 14, 2007, which was towards the end of the major anti-mafia operation referred to as the “Montagna Operation”.

We have chosen the court order associated with the operation Montagna because it contained the largest number of wiretaps and stakeout instances among all court orders we had access to, which allowed us to maximise the size of the dataset.

This operation was concluded in 2007 by the Public Prosecutor’s Office of Messina (Sicily) and was conducted by the Special Operations Unit of the Italian Police (*Reparto Operativo Speciale* (R.O.S.) of the *Carabinieri*, specialized in anti-Mafia investigations).

This particular investigation was a prominent operation focused on two Mafia clans, known as the “*Mistretta*” family and the “*Batanesi*” clan. From 2003 to 2007, these families were found to had infiltrated several economic activities including major infrastructure works, through a cartel of entrepreneurs close to the Sicilian Mafia.

According to the Italian Code of Criminal Procedure, the pre-trial detention order begins with the crimes alleged against individuals. The same individual can be mentioned multiple times in the order: for example, an individual *A* could appear for the first time because she/he is accused of theft; furthermore, *A* could appear together with an individual *B* because both *A* and *B* could be jointly accused of extortion.

Information available in the pre-trial detention order is relevant to the construction of a graph, which falls within the class of the so-called of *co-offending networks* [46]: in a co-offending network, nodes are associated with individuals while edges specify that two individuals (corresponding to the endpoints of that edge) co-participated in perpetrating a crime. The construction of a co-offending network highly depends on the assessments of the public prosecutor, who could classify some activities as criminal ones, while considering other activities as not criminally relevant.

In the light of these reasons, we preferred to focus on graphs that store meetings and phone calls between individuals: these graphs, in fact, are built starting from the observation of individual behaviors and, thus, they do not rely on the legal value that these behaviors could have according to the personal view of the public prosecutor.

The pre-trial detention order reported the composition of both the Mistretta family and the Batanesi clan; judicial proceedings followed a chronological narrative scheme which detailed the illicit activities pursued by the members of the Mistretta clan before the imprisonement of a boss; to preserve anonymity, we will denote such a boss as *x*. The boss *x* has been selected by the most influential Mafia syndicates to settle the conflicts between the Mistretta and the Batanesi families. The conflicts were unleashed from the extortion imposed on local entrepreneurs in the construction of the highway that connects the city of Messina to Palermo.

LEAs relied on the depositions of collaborators of justice, stakeouts and wiretapping.

Meetings involving suspected individuals can be broadly classified as follows: (a) meetings which aim to define the organizational structure of the Mafia syndicate along with its relationships with entrepreneurs and other Mafia syndicates operating in surrounding areas. The boss *x* always attended these meetings and has always been accompanied by at least two trusted men. (b) Meetings involving people who occupied the lowest levels of the Mafia syndicate hierarchy; the purpose of these meetings was, in general, to design illicit activities and, usually, only two people were involved in these meetings. For each one the date of the meeting, the place, and the participants were recorded. Participants to a meeting were identified by a unique identification code. The procedure to build the Meetings network was as follows: (a) each person who participated in at least one meeting corresponds to a node in the network; (b) two subjects in the Meetings network are connected by an edge if both of them attended at least one meeting; (c) edge weights reflect the number of meetings that two individuals jointly attended. Analogously, judicial proceedings recorded phone calls between the individuals under investigation. For each call, the ID of the caller (resp., the ID of the receiver), the time of the call, the duration, and content of the conversation were reported. The procedure to build the Phone Calls network was as follows: (a) each person who made or received at least one call was associated with a node in the network; (b) two nodes in the phone call are connected by an edge if the associated individuals had at least one call; (c) the weight attached to an edge specifies the number of phone calls between the individuals connected by that edge. Observe that we used only text data (from pre-trial detention order) to derive links in both the Meetings and the Phone Calls networks. In addition, we included all actors found in the investigation records, independently of the fact that an actor is actually a member of a Mafia syndicate or not.

### Dataset description

In this section we explain how we have created the datasets (which is fully available on GitHub repository https://github.com/lcucav/criminal-nets/tree/master/disruption) and on Zenodo (https://doi.org/10.5281/zenodo.3938818), generating two criminal networks, directly from Court data. We have previously reported a visual representation of both datasets as undirected and weighted graphs in [35]. In our datasets, each node represents an individual, whereas links show mutual interactions among the individuals. The Meetings dataset, captures the physical meetings among suspects. the Phone Calls dataset refers to phone calls among individuals.

In [35], we introduced a preliminary characterization, whereby we defined the weight distributions and conducted the shortest path length analysis. The weights distribution analysis unveiled a long tail of nodes, with just a few dominating ones. We could observe that a predominant number of links had similar weight (*w* = 1), thus making the distinction between weighted and unweighted graphs negligible after the removal of the better connected nodes. As also confirmed through the shortest path length analysis, the study demonstrated that just a few affiliates tend to be responsible for the highest interaction frequency with a balanced number of intermediates. This is because the clans aim to avoid an overexposure of the bosses (and other prominent affiliates), to minimize police interceptions.

Encouraged by those early results, in the present paper we have moved our attention to understand how LCC varies as a result of police interventions (i.e., the nodes removal process).

The key features of our datasets are summarized in Table 1, including the number of nodes and edges, the maximum weight, the maximum frequency in the affiliates’ interactions, the average degree (neighbours per node), and the highest number of individuals required to connect mobsters (based on all shortest paths in both datasets). We also calculated the APL and the CC of the Meetings and the Phone Calls networks. It is worth noticing that the two datasets have 47 nodes in common and a comment on this overlap is extensively discussed in Subsect. Main limiting factors of our study.

**Table 1:** Characteristics of meetings and phone calls networks.

Parameter	Meetings	Phone Calls
No. Nodes	101	100
No. Edges	256	124
Max. Weight	10	8
Max. Frequency	200	100
Avg. Degree	5.07	2.48
Diameter	7	14
APL	3.308	3.378
CC	0.656	0.105
Common nodes	47	

Indeed, both networks can be treated as either unweighted and weighted networks since, for each pair of individuals, we recorded a coefficient representing the number of times the pair had a meeting (as reported by the police surveillance logs), and made a phone call (reported in police interceptions logs). In SNA terms, these coefficients are known as the strength of the connection between two individuals.

Nodes may belong to different categories. Some nodes represent the leaders (i.e., “bosses”) or the soldiers (i.e., “picciotti”, a Sicilian word which refers to the lowest rank of the Mafia hierarchy) of the criminal organization. Other nodes are associated with individuals (e.g., a fruit seller or a baker) who had one or more calls with members of a Mafia syndicate but are not affiliated with any criminal organization.

The APL and CC values in the Meetings network were then compared with the average values of APL and CC of a random graph *G*^⋆^; in our tests, *G*^⋆^ had the same number of nodes and the same average degree of the Meetings network. In the Meetings network, the APL and CC are equal to 3.308 and 0.656, respectively; in the random graph associated with the Meetings network, we measured an APL and a CC equal to 4.342 and 0.0018, respectively.

We repeated the procedure above for the Phone Call graph and, thus, we generated a random graph with the same number of nodes of the same average degree of the Phone Call graph and we calculated its APL and CC. We observe that the APL and the CC for the Phone Calls network are equal to 3.378 and 0.105, respectively; in the random graph associated with the Phone Calls network, we measured an APL equal to 5.593 and a CC equal to 0.0039. We can therefore conclude that both in the Meetings and Phone Calls networks the CC values are considerably bigger than those found in the corresponding random graph; in contrast, APL values are moderately lower than those found in the corresponding random graph.

In the light of our analysis, both the Meetings and the Phone Calls networks can be regarded as *small world networks* [47]. The high CC coupled with a low average path length favour the information flow among the individuals in the criminal organization and promote their coordination, thus making the organization more effective.

### Experimental method

This section briefly describes the experimental process employed to disrupt the two networks and evaluate the effects of node removal, under different conditions and strategies. Both datasets were used, under both unweighted and weighted conditions. Two node removal strategies have been studied. These are iterative procedures in which the nodes have been removed in decreasing order of their centrality score. After the node removal stage, the LCC size is updated and the process resumes.

Let us denote by *LCC*(*G*) the size of the LCC of a graph *G*. Denote by *G*_*i*_ the graph resulting after the *i*-th iteration of the node removal algorithm, whose size of the LCC is *LCC*(*G*_*i*_). Clearly, the unperturbed graph is *G*_0_, for which we have *LCC*(*G*_0_).

The relative difference between the size of the LCC at the start of the simulation and after the *i*-th iteration (i.e., *i*-th node removal) is given by *ρ*_*i*_ ∈ [0, 1]:

$$\begin{array}{c} \rho_{i}=1-|\frac{LCC\left(G_{i}\right)-LCC\left(G_{0}\right)}{LCC(G_{0})}| \end{array}$$ (9)

Note that *ρ*_0_ = 1 and *ρ*_*n*_ = 0, where *n* is the last iteration (sequential removal).

Both strategies (sequential and block removal) may be summarized as follows:

1. We first compute *LCC*(*G*_0_), i.e., the LCC size for the initial graph *G*_0_.
2. This step depends on the removal strategy. Either the the highest ranking of the remaining nodes (in the sequential strategy), or the the set of the five most influential nodes of the remaining ones (in the block strategy) are removed. Ranks are computed with the current centrality score. The new graph *G*_*i*_, with *i* = 1, 2, …, *n* is obtained (for block removal we have fewer iterations).
3. Compute *LCC*(*G*_*i*_), and calculate *ρ*_*i*_.
4. Steps 2 and 3 are repeated until the graph size can no longer be reduced.

#### Sequential nodes removal

It simulates the scenario in which affiliates are arrested one-by-one by the police.

#### Block nodes removal

It simulates the scenario in which affiliates are arrested during a raid by the police. This strategy is similar to the sequential one, with the main difference being that nodes are removed in blocks of five. The block size depends on the type and scale of the datasets. The fraction of nodes to be removed during block police operations is a reasonable value that takes into account some considerations. In an our previous study [26] in which we obtained a serious reduction of the LCC with only 5% as block size (See Comparison to our earlier works). Moreover, in such a relatively small criminal network larger fractions of block sizes appear unrealistic. This is why, in our case, five has been found to be adequate in terms of number of nodes removed at once.

### Comparison to our earlier works

It is worth highlighting the most significant contributions of the present paper, compared the preliminary data published earlier on [26]. The main differences are as follows:

1. In [26], we introduced a different set of datasets. The first one depicted phone calls among suspected criminals. This was then refined through a different investigative method, i.e., the stakeout, which refers to searching private residences and personal bank information according to a formal search warrant. In this way, we were able to build a second dataset that included crime relationships among mobsters. This second dataset may be considered to be a subset of the first one, including only actual criminals, as classified by the LEAs based on criminal evidence.
  By contrast, the datasets introduced herein have been derived from different investigation methods, namely wiretapping and stakeouts. This comes with the benefit of having two datasets that are only loosely related. In fact, only a fraction of wiretapped individuals also attended face-to-face meetings with other suspects (and vice versa).
  The output of wiretapping and stakeout activities generated two graphs (the Meetings and the Phone Calls networks) with different topological features and with a smaller than expected degree of overlap (i.e., the Meetings and the Phone Calls networks had 100 and 101 subjects respectively, of which only 47 were common to both). The structural analysis of the Meetings and the Phone Calls networks is therefore relevant for identifying which specific features of a Mafia syndicate are properly caught by wiretapping, but are overlooked from stakeout (and vice versa). This kind of analysis was not possible in our previous contribution.
2. Beyond the introduction of new experimental datasets, the present paper has different aims. While in [26], we aimed at disrupting the network (e.g., by enlarging the average path length), herein we seek what may cause the largest drop in LCC size.
3. Unlike our previous work, herein we introduce weighted graphs to describe ties between suspect individuals. Weights attached to graph edges measure the intensity of the tie between two suspects and this information was useful to perform new studies (e.g., the effects due to targeted attacks on weighted graphs), which were missing in [26].
4. In this paper we consider two more centrality measures, the Katz centrality and the Collective Influence, to identify the nodes which should be targeted. For example, the Katz centrality uses the contribution of all paths converging to a node in order to calculate the centrality of that node. Therefore, this provides a substantially different approach to computing node centrality from that provided by Betwenneess and Closeness centrality (exploited in our previous paper), which are based only on the shortest paths.

## Results

In this section the results obtained from our experiments on LCC size are shown. The network analysis is reported next, considering weighted and unweighted graphs scenarios.

### Weighted graphs

**Fig 1:**
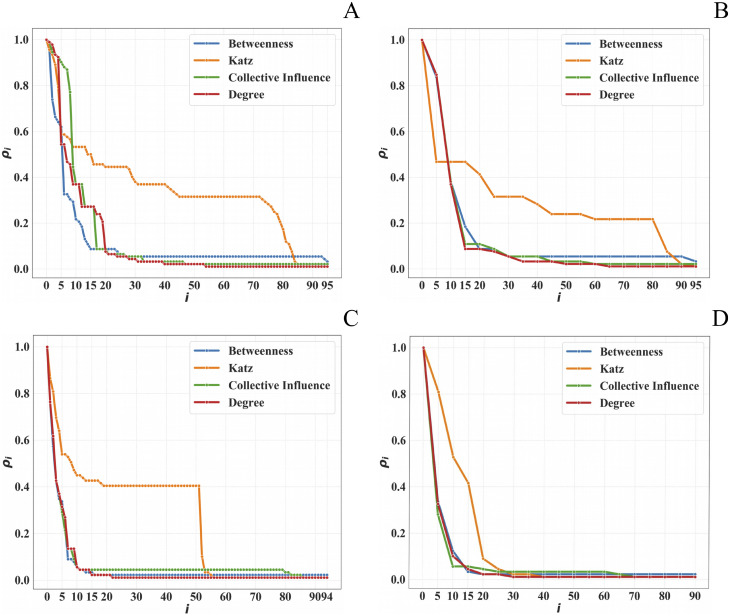
Weighted networks. A: Meetings dataset, sequential node removal strategy. B: Meetings dataset, block node removal strategy. C: Phone Calls dataset, sequential node removal strategy. D: Phone Calls dataset, block node removal strategy.

Fig 1 shows the results obtained for the cases of sequential and block node removal, for both datasets, and including all four centrality metrics. Remarkably, the Katz coefficient (tuned to the default values of *α* = 0.1 and *β* = 1.0) is the least effective one (i.e, the slowest one) at causing the faster LCC size drop, in all eight cases, that are: two datsets (Meetings and Phone Calls), two strategies (sequential and block) and two graph structures (weighted and unweighted).

To understand this result intuitively, we need to look at the way this centrality metric operates. Katz determines the importance of each node based on the number of *walks* that pass through it; but it does not consider their length. Furthermore, shortest path are not considered, hence a walk may visit the same node multiple times. Yet, this is in contrast to how criminals would operate in practice. Affiliates typically prefer to spread the information through a number of intermediaries, to minimize the risk of interception by non-family members. This is consistent with our earlier findings [35]. Ultimately, it would not make sense (and would be unwise) to send the same message multiple times through the same path, which is what Katz would help identifying. Therefore, removing nodes by the highest Katz score would not be a winning strategy.

All the other metrics act better than Katz centrality, and comparably among each other. This happens because of the weights distribution shape [35], which exhibits a long tail of nodes, with just a few dominating ones (Sect. Dataset Description). Thus, after removing the most central nodes (i.e., the first five iterations), the network gets almost totally disconnected and the remaining nodes have the same weight (*w* = 1). Hence, all the metrics focused on either degree (i.e., Degree and Collective Influence) or shortest paths (i.e., Betweenness) follow the same *ρ* drop speed. On the other hand, Katz centrality with its default parameters focuses on walks of undefined lengths, thus producing a slower *ρ* drop.

### Sequential vs block removal

Looking at Fig 1, with the exception of Katz, no significant differences are visible between the two node-removal strategies (i.e., sequential and block). This is somewhat counter-intuitive, since in real life police raids are typically aimed at breaking up the network more effectively. In our case, this result originates from the particular type of the datasets at hand. When constructing the datasets, we did not have access to information about the way criminals reconstructed their communication channels following arrests. Hence, our network is static (i.e., it misses the network reconfiguration data), which is why our analysis is not fully capturing the dynamic aspects that differentiate sequential and block strategies. In network terms, this translates into no differences in terms of LCC size drop as the network is static. On the other hand, significant network re-tuning of nodes importance, due to internal re-organisation of trusted affiliates used to spread messages within and outside the criminal network, would be expected in the case of dynamic graphs (i.e., graph snapshots before and after police operations).

### Weighted vs Unweighted

Considering now the differences between weighted and unweighted graph analysis, we notice that the majority of cases do not pinpoint major differences. This was due to the peculiar way in which weights are distributed in criminal networks (as noted in the *Weighted Graphs* paragraph).

**Fig 2:**
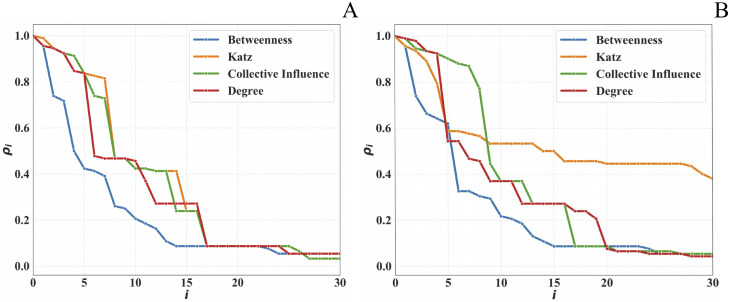
First 30 iterations of the sequential node removal strategy, Meetings dataset. A: Unweighted Graph. B: Weighted Graph.

Nevertheless, interesting differences are visible in the Meetings dataset—sequential node removal (Fig 2). The unweighted case is mostly faster than (although occasionally equivalent to) the weighted case. This is because the weights (i.e., the affiliates’ interaction frequency) are concentrated in very few individuals, with most other weights having *w* = 1. This is also why, with the exception of the initial transient period (involving very few interactions), most algorithms converge to similar values.

### The best centrality metric

Comparing the algorithms in Fig 2, it emerges that Betweeness centrality is by far the most effective centrality index for reducing the size of the LCC of a criminal network.

This result is consistent with literature reports upon criminal networks’ SNA shown in the Introduction, and has an intuitive explanation. Indeed, to avoid being intercepted, members of a criminal networks build their relationships to assure that information flows along the shortest possible paths. In this way, both the Meetings and the Phone Calls network configure themselves as small-world networks with a low average path length and a large CC. The nodes that intercept most of these shortest paths are those having the largest values of Betweenness centrality, and act as intermediaries to assure the quick flow of information from any source to any target in the graph.

**Fig 3:**
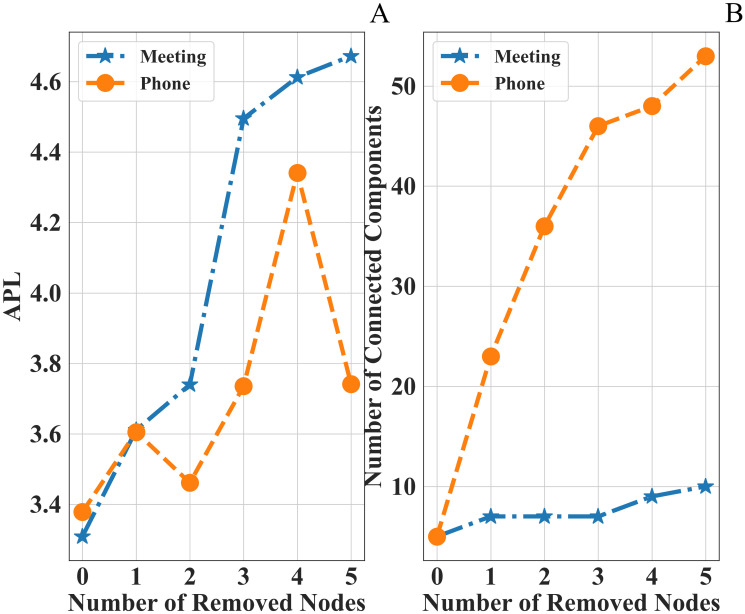
Function variations of the number of removed nodes in the Meetings and phone calls networks. Nodes are prioritized on the basis of their Betweenness centrality. A: Variation of the APL. B: Variation of the number of connected components.

To confirm this intuition, we progressively removed nodes according to their Betweenness centrality and we measured the corresponding variation of APL and the number *n*_*c*_ of connected components (see Fig 3). These plots indicate that the selected removal of nodes amplifies the average distance between any pair of nodes in the Meetings/Phone Calls networks and, simultaneously, it creates an increasing number of disjoint components. A repressive action aimed at removing high Betweenness nodes has, therefore, a devastating impact on network topology because it causes an LCC size drop, as we observed a fast drop in *ρ*. Also, since the Katz centrality prioritizes those nodes crossed by a large number of walks of arbitrary length, it is less effective in detecting the nodes acting as intermediaries, and whose removal reduces the LCC size the most.

Intuitively, Betweenness centrality outperforms the other metrics, thanks to its operation on paths, rather than on individual nodes degree. This is particularly effective in criminal networks that are devised in such a way as to minimize the path length, in order to reduce the risk of police interceptions. Betweeness centrality compromises the most influential paths, leading to a faster drop in *ρ*. This feature is what makes Betweeness somehow opposite to Katz centrality (whose goal is to explore walks).

Collective Influence was the second worst performer after Katz. This is, again, due to its emphasis on node degree instead of path length. Collective Influence is also showing some differences between the weighted and the unweighted processes, exhibiting a lower *ρ* drop in the weighed graph. A possible explanation is that the weighted case identifies as influential nodes not only those with higher weights on the incident links, but also the nodes having high-weight only on immediate neighbours. This could reflect a typical situation in criminal networks, whereby the top-leaders avoid direct exposure and mediate all communications through a single trusted individual (or very few of them). On the other hand, this particular aspect is not detectable in the unweighted analysis.

### Correlation between social capital and human capital

Inspired by the work of [36], we analyzed the correlation between the social capital and the human capital in the criminal organization we are considering.

In Sociology, the social capital denotes a group of tangible and intangible resources such as interpersonal relationships, a shared sense of identity, shared norms and values, trust, cooperation, and reciprocity which are fundamental to assure the functioning of human communities. From our experiments, individuals with high Betweennees contribute significantly to the social capital associated with a criminal organization because they connect the various subgroups composing the criminal organization itself and enable efficient information flow within the organization.

Thereby, the removal of high Betweenness individuals induces a strong reduction in the social capital but is not, however, the greatest loss that a criminal network might suffer from. For instance, in a drug-trafficking organization, individuals with specialized knowledge in chemistry play a pivotal role to carry out the illicit affairs of the organization. Thus, their removal seriously affects the organization. even if those individuals had low Betweenness.

Broadly speaking, the human capital refers to the skills and resources that some components of the organizations have and which play a key role in the functioning and long-term sustainability of the organization. As noted in [36], the most effective repressive actions should simultaneously aim at weakening both the social and human capital of a criminal organization. To this purpose, Villani *et al*. [36] suggested a new index (called CNR—*Criminal Network Resilience*) to assess the resilience of criminal networks, which combines parameters related to social capital, as well as parameters associated with the human capital.

In our study, individuals have three different roles, namely leaders, members, and elements close to the gang but not affiliated with it. Leaders contribute more significantly than others to human capital because they are in charge of making decisions, planning and coordinating criminal actions. In turn, the human capital associated with the members of the gang is greater than those who are close but not affiliated with it. In Table 2 (resp. Table 3) we report the top 10 nodes of the Meetings (resp. Phone Calls) graphs, sorted by decreasing Betweenness values, and their role in the organization. Observe that the node with ID 18 is associated with a leader and has the highest Betweenness, both in the Meetings and in the Phone Calls networks.

**Table 2:** Betweenness centrality values and role (Meetings network).

Position	Node ID	Betweenness centrality	Role
1	18	0.373	Leader
2	47	0.22	Member
3	27	0.159	Leader
4	68	0.126	Member
5	12	0.117	Member
6	25	0.114	Leader
7	29	0.09	Member
8	36	0.072	Member
9	22	0.069	Member
10	11	0.063	Member

**Table 3:** Betweenness centrality value and role (Phone Calls network).

Position	Node ID	Betweenness centrality	Role
1	18	0.418	Leader
2	61	0.282	Member
3	47	0.236	Member
4	29	0.22	Member
5	75	0.096	Member
6	36	0.085	Member
7	27	0.083	Leader
8	68	0.068	Member
9	58	0.06	close
10	70	0.051	close

In addition, many nodes (18, 27, 29, 36, 47, 68) have high centrality, in both the Meetings and in the Phone Calls networks. We underline that both in the Meetings and in the Phone Calls networks there are only two leaders in the top 10 positions, which indicates that there is a weak correlation between social and human capital, and this agrees well with the results of [36].

### Take-home message

In short, our results confirmed the effectiveness of SNA in speeding up the process of reducing the LCC size of criminal networks. Considering our datasets, we could severely affect LCC (with a 70% LCC size drop) by neutralizing less than 5% of the affiliates (either through sequential arrests or police raids). Betweenness centrality performed significantly better than the other three metrics, thanks to its specific focus on paths, rather than simple node degree. This is consistent with the typical operation of criminal networks where information diffuses through the shortest paths and within the organization, to minimize intra-affiliates interactions and, thus, the risk of interception. Therefore, law enforcement interventions should favor path-related centrality metrics (such as Betweenness) instead of other strategies.

## Discussion

Our social capital investigation shows that Betweenness centrality is the most relevant centrality metric, as it causes an effective LCC fragmentation for both networks under scrutiny. Indeed, Betweenness is the only metric (out of the four analysed) that focuses on shortest paths, which reflects the structure of Mafia syndicates. The LCC size drop velocity depends on the capability of centrality metrics to target the appropriate node with the right criterium, in terms of node importance, for the specific network topology. Mafia syndicates topologies are based on trusted affiliates to spread messages (i.e., interact on shortest paths). Thus, it became clear that centrality metrics based on shortest paths led to faster LCC size drops.

Interfering on the paths produces a sensitive LCC size drop among trusted affiliates. The same conclusion has been drawn regardless of weights (to reflect interaction frequencies).

Overall, we could substantially and more rapidly reduce the LCC size by removing the top 5% most influential nodes, computed according to Betweenness score.

This effect is achieved thanks to the network weights distribution (or rather, their concentration within few influential nodes), which leads to a rapid drop in *ρ*. Once the most influential nodes have been removed, the remaining ones are largely characterized by *w* = 1, which makes the weighted and unweighted networks virtually indistinguishable.

Our SNA results can be directly translated onto law enforcement actions, considering that we are now able to efficiently identify the top 5% most trusted affiliates (i.e., the ones typically employed as intermediaries between bosses and the other members).

In turn, we can virtually neutralize the clans’ internal communication infrastructure by getting the trusted affiliates in custody. Intuitively, whenever arrests can be made in block (raids), that would further impair the ability of the criminal communication network to be re-established. However, we have not studied this specific aspect, due to unavailability of necessary data.

The pre-trial detention order is the final outcome of a time-consuming police investigation. Once the inquiry is underway (and even before it has been completed), the actual network is richer than the one derived from the pre-trial detention order. The investigative network includes extra interactions among suspects, which are removed once the judge deems them to be irrelevant. Thus, the final network derived from the original one is partial and misses the data included in the initial investigation. This explains our limits related to the lack of data.

Furthermore, when LEAs inspect on those kinds of criminal networks, most of the time they have prior knowledge thanks to criminal records, even though they may not have a clear picture of the connections between individuals.

Generally speaking, the aim of a “cosca” is to conduct illegal activities (which may vary from place to place and are susceptible to local trends), and to ultimately pursue effective financial benefits. For instance, some clans may focus on drugs, rather than organ trafficking, prostitution, finance, or political influence. Quite commonly, clans pursue multiple activities, which makes it even more difficult to reconstruct the labyrinth of criminal communication networks (and perform SNA thereof).

Our datasets emerged directly from a collection of official juridical acts, and focused on a single criminal activity (the securement of public procurement contracts). This involved a network of entrepreneurs, was confined to a specific geographical area, and captured information over a limited time span. The peculiarity of our networks is that the gang was established in relation to a specific event (procurement of a methanisation process), so it was not a pre-existent organisation. Thus, our dataset captured a relatively simpler snapshot of the complex entanglement of mafia criminals, which constitutes both a strength, and weakness of our study.

Indeed, if LEAs have prior knowledge, then our approach is even more efficient; otherwise, as is the specific network herein considered, two main issues may arise to conduct investigations: (a) *noise*, and (b) different organisational *time scales*. By noise, we mean that LEAs could have too much information (e.g., too many interceptions or surveillance logs, some of which are worthless). By different organisational time scales we indicate that criminals already know how to contact a specific criminal for their illicit purposes (e.g., a sniper), in a way LEAs might not be able to identify. Thus, they have to spend more time to reconstruct the inner relationships by exploring the evidences and, as previously asserted, this is a time consuming process.

On the one hand, the scope of our SNA is limited by the significance and breadth of the datasets at hand. We have mentioned already how a more dynamic analysis of the network could not be done in this case, as for instance, understanding the re-connection ability following events like individual arrests or police raids. Also, we are capturing a single criminal activity in a confined spatio-temporal context. So, it was not possible to detect a broader and more diversified set of communications, such as those taking place in a more complex, multi-activity network. Nor could we detect external communications, such as those involving people who were not directly members of the criminal nets except for entrepreneurs (e.g., politicians, magistrates and businessmen).

On the other hand, the greater specificity of our networks allowed a cleaner analysis, focused on unveiling some hidden communication mechanisms. Having reduced the parameters under scrutiny and the complexity of the system, we could pinpoint a simple, yet effective strategy for unsettling the connectivity of the network through a dramatic drop in the LCC size. This might have not emerged from the analysis of a more complex network. Also, this simpler framework has allowed us to swiftly test out our hypothesis and to obtain reliable results.

This could have been a challenge on a complex network, especially when multiple criminal activities take place in parallel.

### Main limiting factors of our study

We conclude this section by describing the main factors that posed some limits to our study.

1. *Wiretapping is allowed strictly under well-motivated circumstances and for short periods of time, thus limiting the ability of police forces to gather data on Mafia activities*. According to the Italian Code of Criminal Law, LEAs should request the Public Prosecutor an authorization to wiretap conversations, also by means of electronic systems if such an activity is indispensable to prevent crimes. If authorized, LEAs are allowed to wiretap individuals for at most 40 days, which may be extended by up to 20 days. If the Public Prosecutor grants an extension to wiretapping (and, in general, to other surveillance activities), she/he has to produce a written report in which the reasons justifying the prolongation of surveillance activities must be clearly explained. Therefore, LEAs have a limited ability of collecting the data they need to reconstruct the topology of a criminal network and all information produced during non-authorized periods are lost.
2. *Collaborators of justice are often the main source of information for the investigators; yet they cannot always be deemed to be reliable*. Italian LEAs make an extensive use of the depositions of collaborators of justice, i.e., criminals who abandoned a Mafia syndicate and decided to cooperate with criminal justice authorities in order to reveal the composition and organizational structure of a Mafia syindicate. Collaborators of justice are widely considered as a powerful tool in dismantling Mafia syndicates, but their credibility must be carefully checked. In the Montagna operation, there was a collaborator of justice who provided very accurate and detailed information in the early stages of the investigation tasks. Nevertheless, from a certain point onward, that collaborator was no longer considered to be credible and the information he provided was deemed as unreliable.
3. *Mafia leaders avoid using phones to communicate*. Investigations show that the affiliates of a Mafia syndicate are highly suspicious of being tailed by police and, thus, they carefully avoid conversations on cell phones, whenever possible. This implies that the Phone Calls network yields a partial reconstruction of the information flow in a Mafia syndicate. A further consequence arising from the low propensity of some subjects to make/receive phone calls is the low degree of overlap between the Phone Calls network and the Meetings network. In practice, criminals prefer alternative means of communication (e.g., they use intermediaries to convey encrypted messages). Consequently, the Meetings network includes individuals who had never been intercepted by the police forces. In contrast, some eavesdropped individuals had no ties with the Mafia syndicate but are acquainted with some Mafia syndicate members (for instance, because of work, or other family reasons) and they had conversations with them. Therefore, these individuals have never attended any meeting and are excluded from the Meetings network.

## Conclusions

In this work we have used real-world data relating to a “cosca” that operated in Sicily (Italy) during the first decade of the 2000s. Specifically, our two datasets were derived from original juridical acts about two Sicilian clans who sought illegal profits from public procurement proceedings. The main focus was the social capital analysis, investigated by the drop in the size of the LCC of the networks.

To facilitate the reproduction and further extension of our work, we have placed an anonymized version of the datasets and the source code in a public repository (https://github.com/lcucav/criminal-nets/tree/master/disruption) and with citable DOI on Zenodo (https://doi.org/10.5281/zenodo.3938818), including the Meetings dataset (constructed from police stakeouts) and the Phone Calls dataset (derived from police wiretaps). We have also derived weighted and unweighted versions of the datasets.

We have explored mechanisms required for identifying key individuals in the network and, in turn, speed-up the LCC size drop through minimal node removal. We considered two strategies, namely: (i) a sequential node removal approach, and (ii) block removal. The first one simulates the scenario in which the police arrest one “cosca” affiliate at a time. The second one, mimics a police raid. Next, we put to test four different centrality metrics, namely: (i) Degree centrality, (ii) Betweenness centrality, (iii) Katz centrality, and (iv) Collective Influence. The effectiveness of the centrality metrics has been validated trough the *ρ* parameter, which measures the drop of the LCC size, after node removal, compared with the initial LCC size.

The experiments unveiled Betweenness as the most effective metric. Betweenness produced a greater impact in terms of LCC size drop rate, thanks to its prioritization of communication paths, rather than by individual nodes degree. Thus, the resulting optimal strategy was to order nodes by Betweenness centrality score and to remove the nodes by a decreasing order of these scores. This procedure tackled directly the communication mechanisms used by criminal networks, which are designed to minimize the probability of interception by the police.

This work is prone to considerable extensions and adaptations. For one, it would be interesting to conduct a more in-depth comparative study between social and human capital in Mafia associations. In fact, the resilience of criminal networks also depends on the personal qualities and competences of their members. In Network Science, those competences are represented as node labels describing node roles. Therefore, in order to better assess the strength of a criminal organization, we should also look at the human capital endowment.

While we have looked at how to identify the key information intermediaries, another promising angle is the identification of individuals holding highly specialized roles. Criminal organizations are increasingly infiltrating highly specialised activities that require very specific knowledge, skills and competences. For example, pharmacology and chemistry expertise are required for synthetic drug manufacturing processes. The removal of these highly specialised nodes could decisively undermine the resilience of criminal organizations, as such individuals may be extremely difficult to replace.
